# Editorial: Integrated cardiovascular and neural system processes as potential mechanisms of behavior change

**DOI:** 10.3389/fpsyt.2023.1175691

**Published:** 2023-03-21

**Authors:** Marsha E. Bates, David Eddie, Paul M. Lehrer, Robert P. Nolan, Martin Siepmann

**Affiliations:** ^1^Department of Kinesiology and Health, Center of Alcohol and Substance Use Studies, Rutgers University - New Brunswick, New Brunswick, NJ, United States; ^2^Recovery Research Institute and Psychiatry Department, Massachusetts General Hospital and Harvard Medical School, Boston, MA, United States; ^3^Department of Pediatrics, Robert Wood Johnson Medical School, Rutgers, The State University of New Jersey, New Brunswick, NJ, United States; ^4^Behavioural Cardiology Research Unit, University Health Network, Toronto, ON, Canada; ^5^Institute of Medical Science, University of Toronto, Toronto, ON, Canada; ^6^Department of Psychiatry, University of Toronto, Toronto, ON, Canada; ^7^Clinic for Psychotherapy and Psychosomatic Medicine, University Hospital Carl Gustav Carus, Technische Universität Dresden, Dresden, Germany

**Keywords:** heart rate variability, baroreflex, autonomic nervous system, mental health, intervention, emotion regulation

Heart-brain communication drives emotion, thought, and ultimately, behavior. This idea has captured the attention of philosophers, physicians, poets, and physiologists throughout the ages. Recently, there has been an explosion of research highlighting the role of autonomic nervous system regulation, not only in physical health and disease, but also in mental health. As a result of this work, clinical science is increasingly recognizing the value of interventions that address mental health issues at the level of the body as well as the brain (1). Unfortunately, research in this area has remained fragmented, with findings typically disseminated in disorder-specific journals, which diminishes progress in translating promising findings to broadly address mental health problems. Given the global health burden of mental disorders, there is a pressing need to better understand mechanisms of body-brain regulation and develop effective and accessible preventive interventions and treatments, as well as tools to sustain holistic health and recovery.

This Frontiers Special Topic focused on heart-brain interactions that support arousal modulation, emotion regulation, and behavioral flexibility, with a special emphasis on the process of heart rate variability (HRV), its mechanisms of action, and clinical implications of HRV biofeedback and episodic resonance-paced breathing (eRPB; 0.1 Hz/~6 breaths per minute) as mental health intervention tools. We start with a consideration of how the body and brain act as a coherent, adaptive system. Steffen et al. propose an evolutionary model of brain development that highlights the operation of adaptive prediction resulting from interdependent brain networks. Interoceptive and exteroceptive information is used to predict future conditions and needs, and to support optimal adaptation to continuously changing internal and external environments. This aligns with current understanding that the cardiovascular system and the brain continuously signal one another through the baroreflex loop, and that autonomic, cognitive, and emotional regulation share neural circuitry (2).

## HRV as both a biomarker and mechanism

Adult emotional life is profoundly influenced by childhood experience. Childhood trauma is related to increased risk during adulthood for anxiety, depression, substance use disorders, post-traumatic stress disorder (PTSD), and intimate partner violence (3). These disorders often co-occur in adulthood and present with commonalities in emotion dysregulation, changes in neural-circuitry, and diminished cardiovascular regulation. Yet, the mechanisms through which integrated cardiovascular and neural processes affect psychiatric symptomology and emotion regulation remain poorly understood. We addressed this gap both in populations exposed to childhood adversity, and in those that were not. Dale, Kolacz et al. examined the influence of childhood adversity on an index of vagal efficiency and probed direct and mediational relations between childhood maltreatment, anxiety and depression symptoms, and autonomic reactivity to emotional and physical challenges in college students. Their results suggested a potential neural pathway through which early mistreatment shapes cardiovascular regulation and increases risk for anxiety and depression. Kwon et al. examined the link between emotion regulation difficulties, drug use, and resting HRV in young adults. Their study highlighted gender differences that may point to a unique inhibitory-motivational pathway in women who had a history of low-level drug use. Holzhauer et al. explored how stress regulation, stress-induced alcohol craving, and alcohol use in female military veterans may be moderated by progesterone. Increased HRV reactivity to a stress induction predicted higher alcohol craving both in lab and daily assessment. Finally, Fink et al. addressed the mechanisms through which co-occurring behaviors of hazardous alcohol use and intimate partner violence operate. This placebo-controlled, alcohol administration study used an emotion regulation task to investigate respiratory sinus arrythmia in distressed and non-distressed violent partners. Findings suggested that when intoxicated, distressed violent partners may use inefficient emotional regulation strategies when attempting not to respond to provocative partner behavior.

## Clinical applications

One benefit of probing integrated cardiovascular and neural processes is the identification of new intervention targets to interrupt negative affective states, inefficient coping, and unhealthy behaviors. The idea of affecting “neuromodulation” through self-initiated manipulations of respiratory rate is elegant in its simplicity and accessibility. Clinical and laboratory-based research empirically supports HRV biofeedback and eRPB to enhance cognitive, emotional and behavioral regulation in conditions including substance use disorders (4, 5), affective disorders (6, 7), and PTSD (8). Taking these interventions to scale involves determining who is most likely to use them and perceive them as useful. For example, eRPB significantly dampened craving as an adjunct to substance use disorder treatment, however, there were individual differences in usage rates and within-person variations in perceived usefulness across time (9). In this Special topic, Price et al. identified parasympathetic dysregulation, time-varying exposures to different affective triggers for substance use, and the presence of an alcohol use disorder as potential matching variables for the use of arousal modulation to diminish craving.

COVID-19 disease can cause depression, anxiety and impair autonomic functions (10, 11). Dale, Cuffe et al.' large scale survey in the US showed that people diagnosed with COVID-19, and particularly medical providers, experienced increased autonomic reactivity that was associated with prior adversities and current mental difficulties. Autonomic reactivity mediated much of the relationship between prior adversity and current mental health difficulties. The authors discussed their findings in the light of polyvagal theory suggesting that mental health difficulties following COVID-19 infection may result from autonomic hyper-reactivity.

A novel exploration of whether singing at the resonance frequency of cardiovascular system confers beneficial acute physiological and psychological effects similar HRV biofeedback and eRPB was addressed by Tanzmeister et al.. They examined the relative effects of breathing at 0.1 Hz, singing at 0.1 Hz, and breathing and singing at spontaneous rates. While resonance-paced singing and breathing showed comparable signatures of increased HRV in the low frequency range, their effects on sympathetic activation starkly diverged. This points to the need for further understanding of different forms of 0.1 Hz stimulation and the nuances of effects achieved *via* HRV biofeedback, yogic breathing, muscle tension, singing, meditation and other practices wherein our understanding of physiological mechanisms lags far behind evidence of health benefits.

Finally, Jha et al. addressed the role of neurocardiac modulation in performance anxiety of elite pianists who wore cardiovascular monitors pre-, during, and post-performance. Results highlighted the importance of arousal modulation prior to and during performance in contributing to “flow state” (sustained focused attention, task engagement, negotiation of challenge). Their results suggest that interventions to modulate arousal pre-performance may be useful to enhance music performance, and have implications for intervening in subjective stress, autonomic arousal, and disrupted behavior associated with other forms of social anxiety.

## Author contributions

This Research Topic on “*Integrated cardiovascular and neural system processes as potential mechanisms of behavior change*” was initially proposed by invitation to MB and organized with DE. This editorial introduction was led by MB and edited by DE. The editorial team contributed to the content of the published document. All of the editors worked collaboratively to decide which papers were accepted or rejected, and each manuscript was subject to review by one or more of the editors as well as peer reviewers. All authors contributed to the article and approved the submitted version.
